# Is there hip muscle weakness in adults with chronic non-specific low back pain? A cross-sectional study

**DOI:** 10.1186/s12891-023-06920-x

**Published:** 2023-10-07

**Authors:** Gustavo Zanotti Pizol, Katherinne Ferro Moura Franco, Gisela Cristiane Miyamoto, Cristina Maria Nunes Cabral

**Affiliations:** Master’s and Doctoral Program in Physical Therapy, Universidade Cidade de São Paulo, Rua Cesário Galeno 475, São Paulo, 03071-000 Brazil

**Keywords:** Low back pain, Muscular strength, Hip, Trendelenburg, Step down, Dynamometer

## Abstract

**Background:**

Patients with chronic low back pain may present changes in hip muscles. However, there is still limited and controversial evidence of the association between hip muscle weakness and chronic low back pain and whether this weakness can be assessed with functional tests. The purpose of this study was to assess whether there is hip muscle weakness in patients with non-specific chronic low back pain and whether there is an association between the positive Trendelenburg and Step-Down tests and hip muscle strength.

**Methods:**

This cross-sectional study included 40 patients with chronic low back pain and 40 healthy participants, assessed in an outpatient clinic in Vitória, Espírito Santo, Brazil. Muscle strength was measured for the hip abductors, adductors, extensors, internal rotators, and external rotators using isometric manual dynamometry and functional stability was measured by the Trendelenburg and Step-Down tests. Muscle strength was compared using the t test for independent samples and the chi-square test. The association between the tests and strength was performed using a binary logistic regression analysis.

**Results:**

Healthy participants showed a statistically significant greater muscle strength for the right hip abductors (mean difference [MD]: 28.1%, 95% confidence interval [CI]: 9.4 to 46.9), right adductors (MD: 18.7%, 95% CI: 6.2 to 31.2), right internal rotators (MD: 8.7%, 95% CI: 1.5 to 15.8), right extensors (MD: 21.1%, 95% CI: 6.2 to 31.2), left abductors (MD: 30.4%, 95% CI: 11.9 to 49), left adductors (MD: 18.4%, 95% CI: 3.7 to 33.2), and left extensors (MD: 21.6%, 95% CI: 6.6 to 36.5). There was no difference between groups for the positive functional tests, and there was no association between the tests and hip muscle strength.

**Conclusion:**

Patients with chronic low back pain tend to have hip abductors, adductors, and extensors weakness. Furthermore, the functional tests should not be associated to hip muscle strength in patients with chronic low back pain.

## Background

Low back pain is defined as pain located between the region below the last ribs and the gluteal margin, with or without radiation to the lower limbs [1, 2]. In 2015, low back pain was the main cause of years lived with disability in developed and developing countries, reaching 540 million people worldwide [3]. Chronic low back pain (CLBP) is present when symptoms persist for more than 12 weeks [2].

Muscle changes in patients with CLBP extend beyond the trunk muscles, as reported by studies that investigated hip muscle strength in patients with CLBP [4–10]. Four studies found hip muscle weakness in patients with CLBP [4, 6, 7, 10], while two found no weakness in these muscles [5, 9]. In addition to these contrasting results, studies used different approaches when assessing muscle strength. In some studies, the time of contraction during the strength test was not presented [4, 7], while in others there was no application of inelastic resistance for stabilization during data collection [4, 6, 10, 11]. Furthermore, participants’ positions varied during muscle strength data collection while using a manual dynamometer [11–13]. Finally, the majority of the studies did not evaluate hip muscles other than the abductors [4, 6, 7, 10] and did not perform functional tests in their assessments other than the Trendelenburg test [4, 6, 7].

Exercises are the first-line treatment for patients with non-specific CLBP, including segmental stabilization exercises [1, 14]. Understanding which muscles are weaker in these patients can better guide exercise-based treatment. However, dynamic hip stability is not exclusively achieved by hip abductor muscles [15]. The synergism between hip abductors, extensors, and external rotators and their relationship with antagonists are conditions for hip stability [15, 16]. In addition, the gluteus maximus muscle is one of the main synergists for lumbar extension [17], which shows the importance of a complete assessment of hip muscles and not just of the abductors when considering the assessment of lumbopelvic stability in patients with CLBP.

Furthermore, there is a need to investigate whether this strength is associated with positive functional tests that are easy to perform in the clinical environment, such as the Trendelenburg and Step-Down tests. If there is an association between these tests and muscle strength in patients with CLBP, the tests can guide a simpler and less stressful assessment for these patients. If this association does not occur, healthcare providers can focus their assessment on other aspects. Thus, the primary aim of this study was to investigate whether adult patients with non-specific CLBP have changes in strength of the hip abductors, adductors, extensors, and external and internal rotators compared to healthy participants. The secondary aim was to verify whether there is an association between hip strength and the positive Trendelenburg and Step-Down tests.

## Methods

### Study design

This cross-sectional study followed the guidelines of the Strengthening the Reporting of Observational Studies in Epidemiology (STROBE) initiative [18]. This study was prospectively approved by the Research Ethics Committee of Universidade Cidade de São Paulo (approval number 2.895.061).

### Sample

We included 40 patients with non-specific CLBP and 40 healthy participants of both sexes, aged between 18 and 65 years. The sample calculation was performed using an independent t test to detect a 15% difference in hip abductor strength between the groups [19], considering a statistical power of 80%, significance level of 5%, and the inclusion of an additional 30% of participants due to the assessment of other muscle groups.

All participants reported having aerobic activity levels of less than 150 min per week [20], and none were undergoing strength training. The inclusion criteria for patients with non-specific CLBP were pain with a minimum duration of 12 weeks, located in the region below the last ribs to the gluteal margin [2]. The control group was composed of participants without symptoms of pain at the low back, knees, and hips in the last three months [4]. In addition, participants in the control group should answer “yes” to the following questions: “Do you consider yourself healthy for your age?” and “Are you able to participate in normal day-to-day activities for your age group?” [21]. The exclusion criteria for both groups were history of surgery of the lumbar spine, hip, or knees, diagnosis of neurological or rheumatological origin, tumors, or sciatica, amputation of lower limb or part of lower limb, wheelchair use, knee, or hip pain, undergoing physical therapy, and who were unable to complete the questionnaires or understand the instructions for the study tests.

The variables sex, age, and body mass index were paired between groups. Participants were recruited from social media or personal contact. Data collection was carried out from November 2018 to July 2019 at the Running Analysis Laboratory and Physiotherapy in the city of Vitória, Espírito Santo, Brazil.

### Procedures

Participants received information about the study, its objectives, the importance of their participation, and the procedures to which they would be exposed during the assessments. After agreeing to participate, they signed an informed consent form. The participants then underwent a clinical assessment to confirm eligibility for the group of patients with non-specific CLBP or for the control group. All participants answered the Roland-Morris Disability Questionnaire and the Pain Numerical Rating Scale, followed by hip muscle strength assessment via dynamometry. Lastly, the functional tests were performed. Regardless of the results, no one was excluded. All tests were performed on the same day by a single physical therapist. The physical therapist responsible for the evaluations (GZP) was not blind to group allocation. He had seven years of degree in physical therapy and specialization in musculoskeletal physical therapy.

The Roland Morris Disability Questionnaire [22] assessed the participants’ disability due to low back pain. The questionnaire had 24 items and participants must select “yes” or “no” for each statement. The score ranged from 0 to 24 points, and the higher the score, the greater the disability [23]. The Pain Numerical Rating Scale consisted of 11 points, with 0 being “no pain” and 10 being “the worst possible pain” [23]. The Roland Morris Disability Questionnaire [22] and the Pain Numerical Rating Scale [23] have already been translated and adapted to Brazilian-Portuguese and have adequate measurement properties. The score for these two assessment instruments was not used as an eligibility criterion, but rather to characterize the sample.

**Fig. 1:**
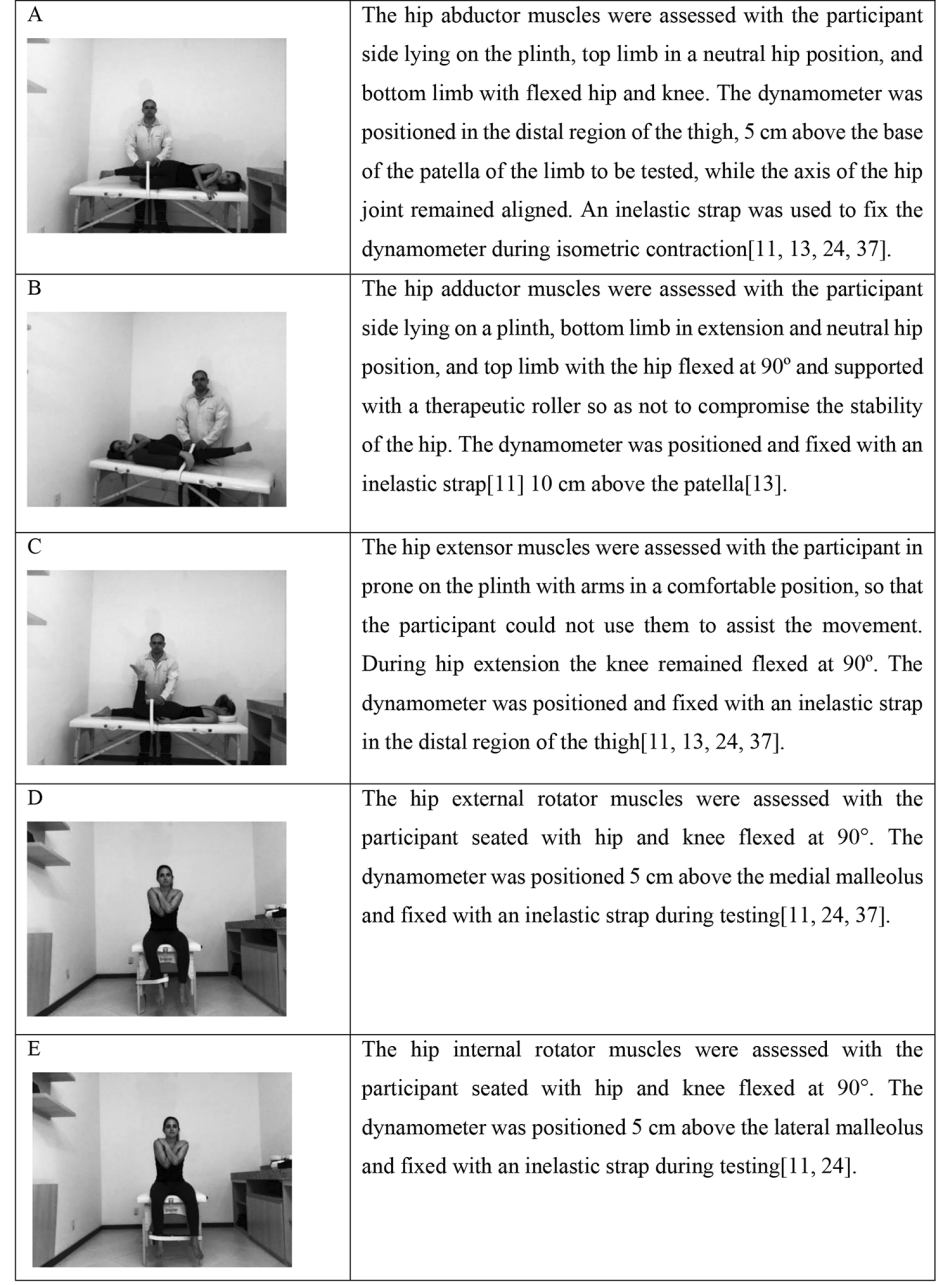
Muscle strength test positions. A: Right hip abductor in left side-lying; B: Right hip adductor in right side-lying; C: Right hip extensor in prone position; D: Right hip external rotator with hip and knee flexed at 90º; E: Right hip internal rotator with hip and knee flexed at 90º

**Fig. 2:**
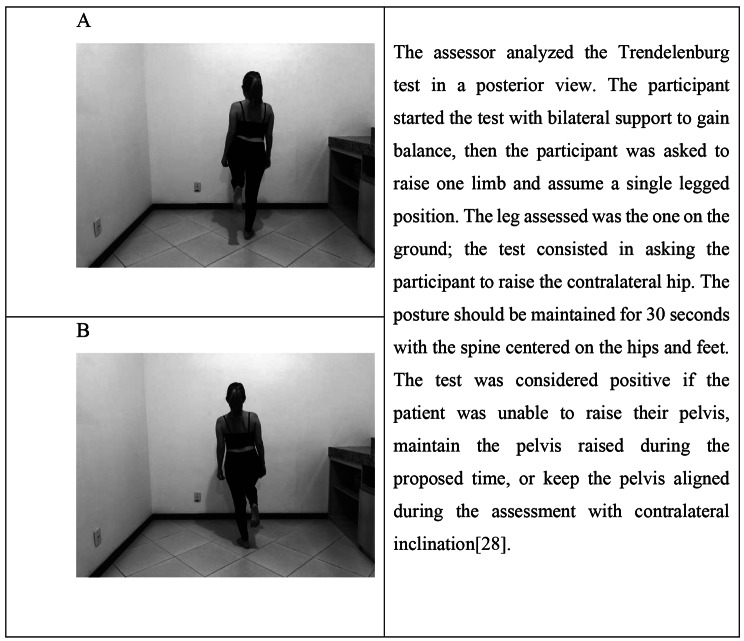
Positions adopted for the Trendelenburg test. A: Right Trendelenburg; B: Left Trendelenburg

**Fig. 3:**
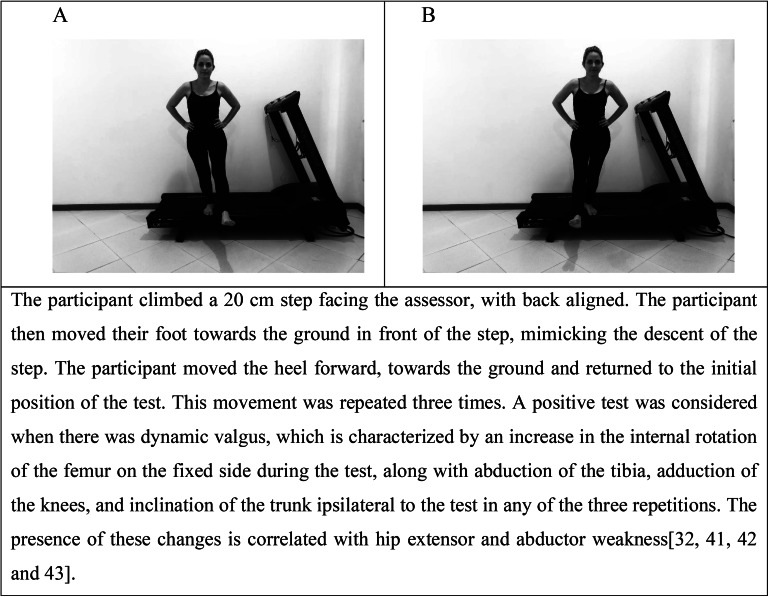
Positions adopted for the Step-Down test. A: Right Step-Down; B: Left Step-Down [32, 41–43]

The Lafayette Instrument® manual dynamometer was used to measure isometric muscle strength. Hip abductor, adductor, extensor, external rotator, and internal rotator muscles were assessed bilaterally, in this order (Fig. 1) [11, 13, 24]. The strength tests were performed by the physical therapist assessor, with excellent intra-examiner reliability for the evaluation of the selected muscles (intraclass correlation coefficient between 0.92 and 0.98). Initially, one submaximal effort test was performed to familiarize participants with the test and the device. Then, two maximum contractions were held for four seconds, and the mean was used for analysis. Participants had an interval of 10 s between contractions for the same muscle group and an interval of 30 s for the next muscle group. If body misalignment was observed during any measurement, a 20-second interval was given and a new measurement was performed [11]. The participant’s dominant side was the first to be assessed. The procedures to assess the Trendelenburg and Step-Down tests are described in Figs. 2 and 3, respectively.

### Statistical analysis

Demographic and clinical data were initially presented through measures of central tendency. Muscle strength was normalized by body weight, using the following formula: (muscle strength in Newtons/body weight in kg)*100 [6, 9]. Then, muscle strength normalized by body weight was compared between the two groups using the t test for independent samples. Alpha value was adjusted using the Benjamini-Hochberg method, which is recommended when multiple comparisons are performed [25]. The between-group comparison for the Trendelenburg and Step-Down tests was performed using the chi-square test. Subsequently, the association between the Trendelenburg and Step-Down tests (dependent variables) and the muscle strength normalized by body weight of the abductors, adductors, extensors, external rotators, and internal rotators (independent variables) was tested using a binary logistic regression analysis. An analysis was conducted for each test separately, using the forced entry method, that is, all independent variables were forced simultaneously in the regression model. The variables with p < 0.05 remained in the model (entry criteria of p ≤ 0.05 and removal criteria of p ≥ 0.10). The assumptions of normality, multicollinearity, and linearity were not violated and were considered present in the occurrence of tolerance < 0.10. A test of normal distribution of the regression models was conducted by plotting both residuals and normal distribution. The level of significance adopted was α < 0.05. The data were analyzed using SPSS version 20.0.0.

## Results

The characteristics of the 80 participants (40 patients with CLBP and 40 healthy participants) are shown in Table 1. The minority of the sample (5%) were smokers, and the time as a smoker ranged from 1 to 12 years and, on average, 7.3 cigarettes were smoked per day. Use of pain medication in the group with non-specific CLBP included anti-inflammatories (7.5%), analgesics (7.5%), and a combination of anti-inflammatories and analgesics (7.5%).

**Table 1:** Demographic and clinical characteristics of the participants

Characteristic	Chronic low back pain group (n = 40)	Control group (n = 40)
Female	26 (65)	27 (67.5)
Age (years)	32 (11.3)	32 (11.5)
Body mass index (Kg/m^2^)	24.5 (3.4)	24 (3.2)
Marital status		
Single	24 (60)	22 (55)
Married	12 (30)	16 (40)
Divorced	4 (10)	1 (2.5)
Widowed	0 (0)	1 (2.5)
Smoker	3 (7.5)	1 (2.5)
Academic level		
Primary education	1 (2.5)	0 (0)
Secondary education	24 (60)	14 (35)
Tertiary education	10 (25)	21 (52.5)
Postgraduation	5 (12.5)	5 (12.5)
Pain intensity (0 to 10 points)	6 (1.6)	0 (0)
Pain duration^§^ (months)	21 (38)	0 (0)
Disability (0 to 24 points)	8.4 (3.9)	0 (0)
Trendelenburg test		
Positive in the right side	12 (30)	8 (20)
Positive in the left side	17 (42.5)	13 (32.5)
Step-Down test		
Positive in the right side	33 (82.5)	35 (87.5)
Positive in the left side	36 (90)	32 (80)

Continuous variables are expressed as mean (standard deviation - SD) and categorical variables are expressed as absolute numbers (percentage)

§Variable expressed as median and interquartile range

There was a statistically significant difference in the strength values for right hip abductors (mean difference: 28.1%, 95% confidence interval [CI]: 9.4 to 46.9), right adductors (mean difference: 18.7%, 95% CI: 6.2 to 31.2), right internal rotators (mean difference: 8.7%, 95% CI: 1.5 to 15.8), right extensors (mean difference: 21.1%, 95% CI: 6.2 to 31.2), left abductors (mean difference: 30.4%, 95% CI: 11.9 to 49), left adductors (mean difference: 18.4%, 95% CI: 3.7 to 33.2), and left extensors (mean difference: 21.6%, 95% CI: 6.6 to 36.5). In all cases, muscle strength for the control group was statistically greater than that of the group with CLBP. The left internal rotators, and both the right and left external rotators showed no statistically significant difference between groups (p > 0.05) (Table 2). For both the Trendelenburg and Step-Down tests, there was no statistically significant difference (p > 0.05).

**Table 2:** Between-group comparison of the muscle strength normalized by body weight (%)

Hip muscles	Chronic low back pain group	Control group	Mean difference (95% CI)	Adjusted p
Right abductors	153.5 (43.2)	181.6 (41)	28.1 (9.4 to 46.9)	0.005*
Left abductors	144.4 (46.8)	174.9 (35.7)	30.4 (11.9 to 49)	0.002*
Right adductors	72.7 (30)	91.3 (26)	18.7 (6.2 to 31.2)	0.005*
Left adductors	74.1 (29.5)	92.6 (36.2)	18.4 (3.7 to 33.2)	0.030*
Right external rotators	54.6 (16.6)	62.1 (15.4)	7.6 (0.4 to 14.7)	0.190
Left external rotators	53.2 (16.1)	57.7 (15.8)	4.6 (-2.6 to 11.6)	2.150
Right internal rotators	65.6 (17.1)	74.2 (15.1)	8.7 (1.5 to 15.8)	0.048*
Left internal rotators	67.4 (19.8)	76.3 (15.4)	8.9 (1 to 16.8)	0.093
Right extensors	87.6 (36)	108.7 (30.3)	21.1 (6.2 to 35.9)	0.010*
Left extensors	81.9 (35.1)	103.4 (32.0)	21.6 (6.6 to 36.5)	0.007*

*Statistically significant difference between groups (p < 0.05)

All data are expressed as mean (standard deviation)

CI: confidence interval

Because the comparison of functional tests between groups did not show any statistically significant difference, all participants were combined for regression analysis. As seen in Table 3, the regression analysis showed no association between functional tests and hip muscle strength (p > 0.05).

**Table 3:** Regression analysis for the dependent variables

	Trendelenburg test - Right side			Step-Down test - Right side		
	Chi Square: 2.950 Pseudo R: 5.4%			Chi Square: 2.988 Pseudo R: 6.4%		
	Beta (SE)	Odds ratio (95% CI)	p	Beta (SE)	Odds ratio (95% CI)	p
Right abductor strength	-0.006 (0.014)	0.994 (0.967 to 1.022)	0.686	0.014 (0.017)	1.014 (0.981 to 1.048)	0.406
Right adductor strength	-0.020 (0.022)	0.981 (0.940 to 1.024)	0.371	-0.011 (0.027)	0.989 (0.938 to 1.042)	0.676
Right extensor strength	0.015 (0.017)	1.015 (0.982 to 1.049)	0.381	-0.008 (0.019)	0.992 (0.955 to 1.030)	0.669
Right internal rotator strength	0.014 (0.027)	1.014 (0.961 to 1.069)	0.613	0.026 (0.034)	1.026 (0.961 to 1.097)	0.440
Right external rotator strength	0.020 (0.031)	1.020 (0.961 to 1.083)	0.519	-0.037 (0.036)	0.964 (0.898 to 1.034)	0.306
Constant	0.456 (1.075)		0.671	-2.029 (1.321)		0.124
	**Trendelenburg test - Left Side**			**Step-Down test - Left side**		
	Chi Square: 1.660 Pseudo R: 2.7%			Chi Square: 6.345 Pseudo R: 13.4%		
	Beta (SE)	Odds ratio (95% CI)	p	Beta (SE)	Odds ratio (95% CI)	p
Left abductor strength	-0.007 (0.013)	0.993 (0.968 to 1.018)	0.584	-0.016 (0.018)	0.984 (0.949 to 1.020)	0.384
Left adductor strength	0.015 (0.018)	1.015 (0.979 to 1.052)	0.415	-0.005 (0.025)	0.995 (0.946 to 1.046)	0.834
Left extensor strength	-0.007 (0.015)	0.993 (0.964 to 1.022)	0.623	0.041 (0.022)	1.042 (0.998 to 1.088)	0.063
Left internal rotator strength	0.014 (0.022)	1.014 (0.971 to 1.058)	0.530	-0.047 (0.033)	0.954 (0.894 to 1.018)	0.158
Left external rotator strength	-0.015 (0.027)	0.985 (0.934 to 1.039)	0.582	0.016 (0.039)	1.016 (0.941 to 1.097)	0.685
Constant	0.819 (0.970)		0.398	-0.808 (1.303)		0.535

Pseudo R: Nagelkerke R Square

The Chi square value of the Hosmer and Lemeshow test for the dependent variable Trendelenburg test in the right side is 4.286, for the dependent variable Step-Down in the right side is 14.346, for the dependent variable Trendelenburg test in the left side is 9.305, and for the dependent variable Step-Down in the left side is 7.121

CI: confidence interval, SE: standard error

## Discussion

The aim of this study was to investigate the existence of changes in strength for hip muscles in patients with non-specific CLBP compared to healthy participants and to investigate whether there is an association between Trendelenburg and Step-Down tests with muscle strength. The results showed that the bilateral strength of hip abductors, adductors, and extensors, and of the right internal rotators was greater in the control group compared to the group with non-specific CLBP. The difference in strength for hip abductors, extensors and adductors between patients with non-specific CLBP and healthy participants was greater than 15%, which is considered a clinically relevant difference in muscle strength [19, 26]. In addition, there was no statistically significant difference between the groups for the positivity of the functional tests, and there was also no association between muscle strength and the Trendelenburg and Step-Down tests.

Four studies, one in the United States [4], two in Canada [6, 7] and one in Iran [10] reported results similar to ours regarding the weakness of hip abductors in patients with CLBP compared to healthy participants. However, the weakness of hip abductors is not a consensus, as other studies showed no difference in strength for hip abductor and extensor muscles in patients with CLBP compared to healthy participants [5, 9]. Some clinical characteristics of participants may suggest the reason for these differences between the results of these studies and ours. The first characteristic is the mean pain intensity of patients with CLBP, which was 1/10 in the Visual Analogue Scale [5]. In our study, patients with CLBP had a mean score of 6/10 in the Pain Numerical Rating Scale. Another characteristic was the varied level of physical activity by participants, as not all participants were sedentary [9, 27]. In another study [28] on street runners with and without CLBP, hip abductors and extensors did not differ between groups. Our study included sedentary participants in both groups, which may have influenced the results.

Regarding body position during the muscle strength tests, other studies used similar positions for hip abductors [4–6, 11, 12, 24], adductors [11], and extensors [4, 11, 12, 24] compared to the positions in the present study. As for the external rotators [12, 29] and internal rotators test position [29], there is still no consensus on whether the hip should be positioned at 0º or 90º of flexion. Krause et al. [12] state that, regardless of hip position, intra-examiner reliability is excellent. In addition, Bloom et al. [29] affirm that there is no difference in strength for external rotator muscles when the test is performed with or without hip flexion. This finding indicates that our choice of positioning to assess hip external rotator strength probably did not influence the result. Another point to be taken into account was the lack of an inelastic strap to stabilize the dynamometer during internal rotator [29] and external rotator testing [12, 29], which differs from our study, and may justify the adoption of different test positions that make it easier to stabilize the isometric contraction manually [12].

The Trendelenburg test, originally described to test the stabilizing muscles of the hip [30], showed no difference between groups, corroborating the results of similar studies [6, 7]. Another study [31] assessed hip abductor strength and stability of seven young men, using a manual dynamometer and the Trendelenburg test, before and after gluteal nerve block. One of the aims was to verify whether the reduction in hip abductor strength would change the result of the Trendelenburg test. A mean reduction of 52% in the hip abductor muscle strength was observed after nerve block. However, the number of patients with a positive result in the Trendelenburg test did not increase. The authors suggest that people capable of producing up to 10% strength in relation to their body weight will not be positive on the Trendelenburg test because the reduction in strength did not result in an increase in positive tests [31]. Finally, Cooper et al. [4] used manual strength tests to identify weakness of the gluteus medius muscle and investigate its relation to the Trendelenburg test in patients with CLBP, classified as unilateral or bilateral. The symptomatic side showed lower gluteus medius strength values and presented with more positive Trendelenburg test [4]. In our study, we did not ask about the side of pain. However, this information would unlikely change our results because there was no difference in the Trendelenburg test between groups. The Step-Down test was also not associated with muscle strength in the current study, even though it is a more challenging test than the Trendelenburg test, with mechanisms associated with weakness of hip abductors, extensors, and external rotators [32], and with different lumbar spine movements in patients with CLBP compared to healthy participants [33]. This result is in line with a previous study [34] that found no correlation between the Step-Down test and the strength of hip abductors and external rotators in healthy participants.

An important highlight of this study is the strength assessment of the five large hip muscle groups (abductors, adductors, extensors, internal rotators, and external rotators) in patients with CLBP and comparison with healthy participants, showing the clinical characteristic of hip muscles with greater clarity. All participants were matched by sex, age, and body mass index, and were sedentary, reducing confounders within our sample. Muscle strength assessment was performed by a single assessor, with excellent reliability, and with the manual dynamometer stabilized with an inelastic strap, given that according to Krause et al. [12], the limitation of the manual test with or without the dynamometer is the strength of the assessor in relation to the strength of the tested muscle group. Furthermore, to prevent participants with more body weight from gaining a greater advantage in strength tests [35], the results of muscle strength testing were normalized by the participants’ body weight [6, 7]. Another highlight of our study was the investigation of the association of the Step-Down test with hip muscle strength in patients with CLBP. Finally, we can also highlight the clear methodology, allowing assessments to be replicated in clinical practice by healthcare providers with different levels of clinical experience.

The absence of an association between hip muscle strength and the functional tests allows the optimization of clinical assessments. Future studies could assess bioimpedance to observe how the amount of muscle mass could influence the results for muscle strength and functional tests. In addition, we would recommend investigating whether other tests that aim to measure hip muscle strength, such as the Y-Balance test [16], would be better correlated with muscle strength. Prospective studies can assess whether hip muscle weakness may be a risk factor for low back pain, as well as to investigate whether the increase in strength of these muscles can alter the clinical condition of patients with CLBP, which is not yet well established in the literature [17, 36].

Although the present study did not aim the treatment of patients with CLBP, our data may influence decision-making in interventions. Patients with recurrent low back pain episodes tend to evolve with physical deconditioning [37]. So, the health professional should assess the strength of hip muscles of patients with CLBP [1, 2] as a first step to treatment. Additionally, looking forward a strategy to prevent CLBP is very important, as CLBP is prevalent worldwide [38] and usually the patients decrease their activities in some point [37]. The results of this study showed that Trendelenburg and Step-Down tests do not associate with hip muscle strength in patients with CLBP neither healthy participants. Therefore, the assessment of hip muscle strength should be performed with dynamometer, highlighting that the hand-held dynamometer shows a good reliability with the isokinetic dynamometer [19].

### Limitations

However, this study presents some limitations. The study sample consists of relatively young participants (32 years old), with a mean age below the mean age of those most affected by low back pain (between 40 and 80 years old) [39]. All participants were sedentary, but with a normal body mass index, which is not always found in patients with low back pain [40]. Thus, this can make it difficult to generalize the results for a population with different characteristics. The Trendelenburg and Step-Down tests were assessed by a single assessor, who was not blind to group allocation; however, the lack of blinding by the assessor probably did not interfere in the results. Another study [7] using 2D cameras to assess the Trendelenburg test and a manual dynamometer to assess hip abductor strength found no difference between the Trendelenburg test in patients with CLBP compared to healthy participants. Nevertheless, our results related to the absence of relationship between hip abductor strength and the Trendelenburg test are in agreement with those of the study by Penney et al. [6], in which the assessor was blinded.

## Conclusion

Hip abductors, adductors, and extensors are weaker in patients with non-specific CLBP compared to healthy participants. This difference is clinically relevant; however, there is no difference between groups in the results for the Trendelenburg and Step-Down functional tests. Moreover, there is no association between hip muscle strength and the results of the Trendelenburg and Step-Down tests.

## Data Availability

All data used in this study are available upon request and may be reused for individual patient data analysis. Pizol is responsible for the data (gustavo.pizol@gmail.com).

## Abbreviations

CLBP: Chronic low back pain
STROBE: Strengthening the Reporting of Observational Studies in Epidemiology
CI: Confidence interval

## References

[1] National Guideline C. National Institute for Health and Care Excellence: Clinical Guidelines. Low Back Pain and Sciatica in Over 16s: Assessment and Management. London: National Institute for Health and Care Excellence (UK), Copyright. (c) NICE, 2016.; 2016.

[2] Delitto A, George SZ, Van Dillen L, Whitman JM, Sowa G, Shekelle P (2012). Low back pain. J Orthop Sports Phys Ther.

[3] Disease GBD, Injury I, Prevalence C. Global, regional, and national incidence, prevalence, and years lived with disability for 310 diseases and injuries, 1990–2015: a systematic analysis for the global burden of Disease Study 2015. Lancet (London, England). 2016;388(10053):1545–602. 10.1016/S0140-6736(16)31678-6 PMC5055577 27733282

[4] Cooper NA, Scavo KM, Strickland KJ, Tipayamongkol N, Nicholson JD, Bewyer DC (2016). Prevalence of gluteus medius weakness in people with chronic low back pain compared to healthy controls. Eur Spine J.

[5] Sutherlin MA, Hart JM (2015). Hip-abduction torque and muscle activation in people with low back pain. J Sport Rehabil.

[6] Penney T, Ploughman M, Austin MW, Behm DG, Byrne JM (2014). Determining the activation of gluteus medius and the validity of the single leg stance test in chronic, nonspecific low back pain. Arch Phys Med Rehabil.

[7] Kendall KD, Schmidt C, Ferber R (2010). The relationship between hip-abductor strength and the magnitude of pelvic drop in patients with low back pain. J Sport Rehabil.

[8] Nelson-Wong E, Gregory DE, Winter DA, Callaghan JP (2008). Gluteus medius muscle activation patterns as a predictor of low back pain during standing. Clin Biomech (Bristol Avon).

[9] Pilz B, Vasconcelos RA, Teixeira PP, Mello W, Oliveira IO, Ananias J (2020). Comparison of hip and Lumbopelvic Performance between Chronic Low Back Pain Patients Suited for the functional optimization Approach and healthy controls. Spine.

[10] Arab AM, Nourbakhsh MR (2010). The relationship between hip abductor muscle strength and iliotibial band tightness in individuals with low back pain. Chiropr Osteopat.

[11] Magalhaes E, Fukuda TY, Sacramento SN, Forgas A, Cohen M, Abdalla RJ (2010). A comparison of hip strength between sedentary females with and without patellofemoral pain syndrome. J Orthop Sports Phys Ther.

[12] Krause DA, Neuger MD, Lambert KA, Johnson AE, DeVinny HA, Hollman JH (2014). Effects of examiner strength on reliability of hip-strength testing using a handheld dynamometer. J Sport Rehabil.

[13] Bandinelli S, Benvenuti E, Del Lungo I, Baccini M, Benvenuti F, Di Iorio A (1999). Measuring muscular strength of the lower limbs by hand-held dynamometer: a standard protocol. Aging (Milan Italy).

[14] Qaseem A, Wilt TJ, McLean RM, Forciea MA, Denberg TD, Barry MJ (2017). Noninvasive treatments for Acute, Subacute, and chronic low back Pain: a clinical practice Guideline from the American College of Physicians. Ann Intern Med.

[15] Retchford T, Crossley KM, Grimaldi A, Kemp JL, Cowan SM (2013). Can local muscles augment stability in the hip? A narrative literature review. J Musculoskelet Neuronal Interact.

[16] Polkowski GG, Clohisy JC (2010). Hip biomechanics. Sports Med Arthrosc Rev.

[17] Kankaanpää M, Laaksonen D, Taimela S, Kokko SM, Airaksinen O, Hänninen O (1998). Age, sex, and body mass index as determinants of back and hip extensor fatigue in the isometric Sørensen back endurance test. Archives of Physical Medicine and Rehabilitatio Arch Phys Med Rehabil n.

[18] Von Elm E, Altman DG, Egger M, Pocock SJ, Gøtzsche PC, Vandenbroucke JP (2014). The strengthening the reporting of Observational Studies in Epidemiology (STROBE) Statement: guidelines for reporting observational studies. Int J Surg.

[19] Chamorro C, Armijo-Olivo S, De la Fuente C, Fuentes J, Javier Chirosa L (2017). Absolute reliability and concurrent validity of hand held Dynamometry and Isokinetic Dynamometry in the hip, knee and Ankle Joint: systematic review and Meta-analysis. Open Med (Warsaw Poland).

[20] Organization WH. Global recommendations on physical activity for health. World Health Organization; 2010. 26180873

[21] McKay MJ, Baldwin JN, Ferreira P, Simic M, Vanicek N, Hiller CE (2016). 1000 norms project: protocol of a cross-sectional study cataloging human variation. Physiotherapy.

[22] Costa LO, Maher CG, Latimer J, Ferreira PH, Pozzi GC, Ribeiro RN (2007). Psychometric characteristics of the brazilian-portuguese versions of the functional rating index and the Roland Morris disability questionnaire. Spine.

[23] Costa LO, Maher CG, Latimer J, Ferreira PH, Ferreira ML, Pozzi GC (2008). Clinimetric testing of three self-report outcome measures for low back pain patients in Brazil: which one is the best?. Spine.

[24] Bolgla LA, Earl-Boehm J, Emery C, Hamstra-Wright K, Ferber R (2015). Comparison of hip and knee strength in males with and without patellofemoral pain. Phys Ther Sport.

[25] Glickman ME, Rao SR, Schultz MR (2014). False discovery rate control is a recommended alternative to Bonferroni-type adjustments in health studies. J Clin Epidemiol.

[26] Ireland ML, Willson JD, Ballantyne BT, Davis IM (2003). Hip strength in females with and without patellofemoral pain. J Orthop Sports Phys Ther.

[27] Nadler Nadler SF, Malanga GA, Feinberg JH, Prybicien M, Stitik TP, DePrince M (2001). Relationship between hip muscle imbalance and occurrence of low back pain in collegiate athletes: a prospective study. Am J Phys Med Rehabil.

[28] Cai C, Kong PW (2015). Low back and lower-limb muscle performance in male and female recreational runners with chronic low back pain. J Orthop Sports Phys Ther.

[29] Bloom N, Cornbleet SL (2014). Hip rotator strength in healthy young adults measured in hip flexion and extension by using a hand-held dynamometer. Pm r.

[30] Hardcastle P, Nade S (1985). The significance of the Trendelenburg test. J Bone Joint Surg Br.

[31] Kendall KD, Patel C, Wiley JP, Pohl MB, Emery CA, Ferber R (2013). Steps toward the validation of the Trendelenburg test: the effect of experimentally reduced hip abductor muscle function on frontal plane mechanics. Clin J Sport Med.

[32] Powers CM (2010). The influence of abnormal hip mechanics on knee injury: a biomechanical perspective. J Orthop Sports Phys Ther.

[33] Hemming R, Sheeran L, van Deursen R, Sparkes V (2018). Non-specific chronic low back pain: differences in spinal kinematics in subgroups during functional tasks. Eur Spine J.

[34] Hollman JH, Ginos BE, Kozuchowski J, Vaughn AS, Krause DA, Youdas JW (2009). Relationships between knee valgus, hip-muscle strength, and hip-muscle recruitment during a single-limb step-down. J Sport Rehabil.

[35] Jaric S (2002). Muscle strength testing: use of normalisation for body size. Sports Med.

[36] Kendall KD, Emery CA, Wiley JP, Ferber R (2015). The effect of the addition of hip strengthening exercises to a lumbopelvic exercise programme for the treatment of non-specific low back pain: a randomized controlled trial. J Sci Med Sport.

[37] Bousema EJ, Verbunt JA, Seelen HAM, Vlaeyen JWS, Knottnerus AJ (2007). Disuse and physical deconditioning in the first year after the onset of back pain. Pain.

[38] Global regional (2016). National incidence, prevalence, and years lived with disability for 310 diseases and injuries, 1990–2015: a systematic analysis for the global burden of Disease Study 2015. Lancet.

[39] Hoy D, Bain C, Williams G, March L, Brooks P, Blyth F (2012). A systematic review of the global prevalence of low back pain. Arthritis Rheum.

[40] Walsh TP, Arnold JB, Evans AM, Yaxley A, Damarell RA, Shanahan EM (2018). The association between body fat and musculoskeletal pain: a systematic review and meta-analysis. BMC Musculoskelet Disord.

[41] Bell-Jenje T, Olivier B, Wood W, Rogers S, Green A, McKinon W (2016). The association between loss of ankle dorsiflexion range of movement, and hip adduction and internal rotation during a step down test. Man Ther.

[42] Østerås B, Østerås H, Torstensen TA, Vasseljen O. Dose–response effects of medical exercise therapy in patients with patellofemoral pain syndrome: a randomised controlled clinical trial. Physiotherapy. 2013;99(2):126–31. 10.1016/j.physio.2012.05.009 23219636

[43] Loudon JK, Wiesner D, Goist-Foley HL, Asjes C, Loudon KL (2002). Intrarater reliability of functional performance tests for subjects with patellofemoral pain syndrome. J Athl Train.

